# Nasopharyngeal fiberendoscopy in children: a survey of current Italian pediatric otolaryngological practices

**DOI:** 10.1186/s13052-016-0234-y

**Published:** 2016-03-01

**Authors:** Sara Torretta, Paola Marchisio, Giovanni Succo, Pasquale Capaccio, Lorenzo Pignataro

**Affiliations:** Otolaryngology Unit, Department of Clinical Sciences and Community Health, University of Milan, Fondazione IRCCS Ca’ Granda Ospedale Maggiore Policlinico, Via F. Sforza 35, 20122 Milan, Italy; Pediatric Highly Intensive Care Unit, Department of Pathophysiology and Transplantation, University of Milan, Fondazione IRCCS Ca’ Granda Ospedale Maggiore Policlinico, Milan, Italy; Academic Oncologic Department, Otolaryngology Service, University of Turin, San Luigi Gonzaga Hospital, Orbassano Turin, Italy

**Keywords:** Endoscopy, Nasopharynx, Children, Adenoids, Otolaryngology

## Abstract

**Background:**

Nasopharyngeal fiberendoscopy (NFE) is the gold standard diagnostic procedure for adenoidal disease, but there is no consensus concerning the optimal technical approach. The aim of this study was to investigate the attitudes of Italian otolaryngologists towards diagnostic NFE in children, and the most widely used methods.

**Methods:**

Nine hundred randomly selected members of the two largest Italian otolaryngological scientific societies were e-mailed an anonymous web-based questionnaire containing 29 multiple-choice items regarding their opinions about, and use of NFE in children.

**Results:**

Questionnaires were returned by 764 clinicians (84.9 %). About 75 % declared they used NFE, but 35 % said they preferred alternative diagnostic methods. Most of the respondents considered NFE safe, but more than 80 % judged it to be poorly or only fairly well tolerated. Almost all declared that they generally use flexible, small-diameter instruments, with the patient seated on a chair or a parent’s lap; 65 % use gentle restraining methods. Fewer than 50 % reported using a standardised hypertrophy grading system.

**Conclusion:**

Italian otolaryngologists have a generally positive attitude towards using NFE in children. However, some have reservations, and there was no unanimous agreement concerning how it should be done. Given the medical advantages of NFE, it is essential to clarify the many still controversial aspects of the procedure by means of comparative studies and educational programmes.

## Background

Nasal obstruction due to recurrent or chronic adenoid-related nasopharyngeal and middle ear infections is frequent in children [1], and often requires an otolaryngological examination in order to assess whether the size of the adenoids and their possible lateral extension are reducing the patency of the ostium of the Eustachian tube, and evaluate the need for surgical treatment.

This can be done by means of nasopharyngeal fiberendoscopy (NFE), a mirror examination with posterior rhinoscopy, a lateral neck roentgenogram, or a standardised clinical questionnaire [2–4]. However, NFE is the gold standard for assessing the volume and surface of the adenoids in children with suspected adenoidal disease [5–7] because it is minimally invasive and repeatable, does not expose patients to harmful ionising radiation, and allows direct inspection of the nasopharynx with complete visualisation of the adenoids, thus making it possible to grade hypertrophy and investigate possible choanal or ostial obstruction [5]. It is also a useful means of dynamically evaluating the entire nasal district, including the ostiomeatal complex and sphenoethmoidal recess, and investigating velopharyngeal closure during speech. This makes it possible to identify or exclude any concomitant nasal or rhinosinusal processes, including allergic rhinitis, nasal polyposis, chronic rhinosinusitis, choanal atresia or velar insufficiency, and allows precise surgical stratification in the case that medical treatment fails.

NFE should therefore be considered a first-level procedure in every otolaryngological facility that has pediatric patients [8]. However, some clinicians are still reluctant because it is not always easy to carry out in the case of younger children and, furthermore, there is no consensus concerning how it should be performed (particularly in relation to the type of instruments to be used) or the most appropriate technical approach to children of different ages or with different diseases. Consequently, it is not clear how, or how extensively NFE is used in everyday practice, particularly in the case of younger children.

The main aim of this study was to evaluate the attitudes of Italian otolaryngologists towards using NFE to diagnose adenoidal diseases in children, and verify the methods actually used in routine clinical practice.

## Methods

### Study design

This cross-sectional survey of the pediatric use of NFE by a representative sample of otolaryngologists belonging to the Italian Society of Otolaryngology and Head and Neck Surgery (SIOeChCF) and the Italian Society of Pediatric Otolaryngology (SIOP) was carried out between September 2012 and May 2013. The study was approved by our local ethics committee of University of Milan.

### Study population

An anonymous questionnaire asking about opinions and practices relating to the pediatric use of NFE was sent to 900 Italian otolaryngologists whose e-mail addresses were selected by means of a computer-based randomisation list.

### Questionnaire design and administration

The web-based questionnaire, which was anonymous but coded in order to be able to identify non-responders and ensure the elimination of multiple responses, was conceived by the first author (ST) and drawn up in collaboration with the co-authors before being pilot tested on a sample of 20 otolaryngologists in Milan, Italy. It required about 10 min to complete and guided the respondents through multiple-choice items divided into two main sections: one concerning their personal and demographic data (including gender, and the years of birth, graduation and specialisation); the other consisted of 29 items concerning their attitudes towards using diagnostic NFE in children, and the methods they use in routine clinical practice.

### Statistical analysis

The data were descriptively analysed to assess the prevalence and distribution of all the variables. The continuous variables were expressed as mean values and standard deviations (SDs), and the categorical variables as absolute numbers and percentages. The categorical variables were dichotomously analysed at multiple levels. The Kruskal-Wallis equality-of-populations rank test and Fisher’s exact test were used to determine whether attitudes toward NFE and the way it was carried out were related to the demographic data. After adjusting for the main confounders, univariate and multivariate logistic regression models were used to compute odds ratios (ORs) and standard errors (SEs) and 95 % confidence intervals (95 % CIs) in order to measure the strength of the associations. Statistical significance was set at *p* = 0.05. The data were analysed using STATA 10.0 software (StataCorp, College Station, TX).

## Results

Questionnaires were returned by 764 of the 900 otolaryngologists (84.9 %), most of whom were males (589; 77.1 %), aged >50 years (395; 51.7 %), worked in northern Italy (455; 59.5 %), and practised in a hospital setting (397; 52.0 %) (Table 1).

**Table 1:** Demographic characteristics of the otolaryngologists returning completed questionnaires

Demographic characteristics		No. of respondents	Percent
Total number		764	
Males		589	77.1
Age, years			
	≥50	395	51.7
	36–50	322	42.1
	≤35	47	6.2
No. of otolaryngologists working in Northern Italy		455	59.5
Work setting			
	Hospital	397	52.0
	University	70	9.2
	Private practice	297	38.8
No. of years since graduation			
	≥31	159	20.8
	20–30	314	41.1
	11–19	164	21.5
	≤10	127	16.6
No. of years since specialising in otolaryngology			
	≥31	67	8.7
	20–30	302	39.5
	11–19	189	24.8
	≤10	206	27.0

Table 2 shows their attitudes towards NFE. About 75 % of the respondents declared that they used NFE, but 35 % said they still preferred alternative diagnostic procedures to investigate adenoidal disease. About 65 % chose a clinical evaluation (history of recurrent nasopharyngeal and/or middle ear infection and/or sleep disordered breathing, perceived nasal obstruction, speech hyponasality, and the proportion of oral breathing) as the elective alternative method. More than half said they used NFE only in children aged 3–8 years, and nearly 60 % declared that they were able to complete the examination in more than 95 % of children. NFE was considered safe by most of the respondents as 94 % declared the occurrence of untoward effects (mainly nasal bleeding) in fewer than 5 % of cases; however, more than 80 % judged that it was poorly (about 52 %) or only fairly well tolerated (about 32 %). The majority had a positive opinion concerning the usefulness of NFE, and as many as 68 % defined it “a generally well-tolerated, minimally invasive examination that can be used in most children; very useful in clinical practice”.

**Table 2:** Otolaryngologists’ attitudes towards nasopharyngeal fiberendoscopy (NFE) in children

Parameter	Possible answers	No. of respondents	Percent
Used to using NFE		576	75.4
Used to using alternative diagnostic tests			
	Clinical evaluation	175/269	65.0
	Standardised questionnaires	51/269	19.0
	Posterior rhinoscopy	34/269	12.7
	Nasopharyngeal X-ray	9/269	3.3
Age of patients in whom NFE is considered feasible			
	All pre-school years	104	13.5
	>3 years	235	30.8
	3–8 years	425	55.7
Indications for NFE			
	Nasal obstruction	33	4.3
	Adenoidal facies	15	2.0
	Recurrent or chronic middle ear disease	62	8.1
	Rhinosinusitis	26	3.4
	All of the above	628	82.2
Indications for in-patient NFE			
	Children with a genetic syndrome	42	5.5
	Uncooperative children	65	8.5
	Children aged <18 months in whom severe disease is highly suspected	84	11.0
	All of the above	283	37.0
	Children with genetic syndrome or aged <18 months in whom severe disease is highly suspected	290	38.0
Percentage of children in whom NFE is not considered feasible			
	≤5 %	448	58.6
	6–24 %	249	32.6
	25–50 %	46	6.0
	49–74 %	11	1.5
	≥75 %	10	1.3
Percentage of children experiencing untoward effects			
	≤5 %	718	94.0
	6–25 %	44	5.8
	26–50 %	2	<1
Untoward effects			
	Nasal bleeding	579	75.8
	Traumatic lesions	64	8.4
	Syncope	90	11.8
	Desaturation	21	2.7
	Other	10	1.3
Tolerability			
	None	79	10.3
	Poor	401	52.5
	Fair	241	31.6
	Good	37	4.9
	Excellent	7	<1
Final evaluation of NFE			
	A generally well-tolerated, minimally invasive examination that can be used in most children; very useful in clinical practice	518	67.8
	A not always well-tolerated, minimally invasive examination that should only be used in the case of strong diagnostic suspicion; moderately useful in clinical practice	238	31.1
	A poorly tolerated invasive examination that should only be used in selected cases; not very useful in clinical practice	8	1.1

Table 3 shows the methods of use. Almost all of the clinicians generally use flexible, small- diameter instruments, with the patient seated on a chair or a parent’s lap. About 65 % said they use gentle restraint (the method preferred by about 82 % is to have the patient sitting on a parent’s lap “with legs held between the thighs of the parent, who holds the child’s wrists over the abdomen with one hand and the child’s head against his or her chest with the other”). About one-third said that they did not use any topical drug before performing NFE, whereas 30 % said they used local vasoconstrictors.

**Table 3:** Otolaryngologists’ methods of carrying out nasopharyngeal fiberendoscopy (NFE) in children

Parameter	Possible answers	No. of otolaryngologists	Percent
Recommended type of endoscope			
	Flexible	720	94.3
	Rigid	44	5.7
Recommended endoscope diameter			
	About 2 mm	312	40.9
	About 3 mm	369	48.3
	About 4 mm	83	10.8
Recommended sterilisation			
	Disposable sheaths	574	75.2
	Disposable towels	140	18.3
	Antiseptic solutions	50	6.5
Use of endoscope connected to a video recorder/monitor set			
	Yes	588	76.9
	No	176	23.1
Method of removing nasal secretions before NFE			
	None	184	24.1
	Urging child to blow his/her nose	211	27.6
	Helping child to blow his/her nose	117	15.3
	Nasal saline irrigation	44	5.7
	Aspiration	208	27.3
Recommended position for NFE			
	Seated (alone or on parent’s lap)	714	93.5
	Lying on back	50	6.5
Need for restraint			
	Never	221	28.9
	Only younger children	495	64.8
	Always	48	6.3
Recommended method of restraint			
	Holding head gently	67/543	12.4
	Sitting on a parent’s lap^a^	443/543	81.6
	Lying on back wrapped in a sheet	33/543	6.0
Restrainers			
	Only parents	202/543	37.3
	Health workers, if parents unable to cooperate	341/543	62.7
Local pre-medication			
	None	244	32.0
	Vasoconstrictors	231	30.2
	Anesthetic	169	22.1
	Lubricating ointment	120	15.7
Frequency of bilateral NFE			
	Never	20	2.7
	Sometimes	403	52.7
	Always	341	44.6
First anatomical landmark assessed			
	Adenoids and nasopharynx	562	73.7
	Ostiomeatal complex	200	26.3
Frequency of evaluation of anatomical structures other than adenoids during NFE			
	Never	10	1.3
	Sometimes	348	45.5
	Always	406	53.2
How adenoidal hypertrophy is graded			
	Percentage of choanal obstruction	252	33.0
	Percentage of choanal obstruction and patency of Eustachian tube orifice	417	54.5
	Adenoidal hypertrophy: yes/no	7	1.0
	Choanal obstruction: yes/no	88	11.5
Standardised classification for grading adenoidal hypertrophy			
	None	400	52.4
	Cassano’s classification [5]	275	36.0
	Parikh’s classification [9]	65	8.5
	Other	24	3.1

^a^with legs held between the thighs of the parent, who holds the child’s wrists over the abdomen with one hand and the child’s head against his or her chest with the other

More than half declared that they graded adenoidal hypertrophy on the basis of the percentage of adenoid-induced choanal obstruction and the patency of the Eustachian tube, and fewer than 50 % that they used a standardised grading system (mainly Cassano’s [5], which was chosen by 36 % of the respondents).

Table 4 shows the significant associations between the otolaryngologists’ attitude towards the pediatric use of NFE and their demographic data. The use of NFE in clinical practice was apparently influenced by gender, age, and geographical working area because the most frequent users were male clinicians aged <50 years working in northern Italy. However, logistic multivariate analysis showed that only gender adjusted for geographical working area remained significantly associated with the routine use of NFE (OR = 2.4, SE = 0.6, 95 % CI = 1.5-3.7; *p* < 0.001 for males).

**Table 4:** Otolaryngologists’ attitudes towards nasopharyngeal fiberendoscopy (NFE) in children by demographic variables (only statistically significant relationships)

Parameters	Demographic variables			*P*-value
Used to performing NFE				
	Gender	% of males	% of females	0.004
		77.7	66.2	
	Age	% aged ≥50 years	% aged <50 years	0.002
		70.6	80.3	
	Geographical working area	% in northern Italy	% working in southern Italy	0.050
		78.0	71.8	
Used to performing alternative diagnostic tests				
	Gender	% of males	% of females	0.013
		33.5	44.8	
	Age	% aged ≥50 years	% aged <50 years	<0.001
		45.1	28.6	
	Years since specialisation	% specialised for ≥20 years	% specialised for <20 years	0.043
		41.2	33.7	
Used to performing NFE regardless of patient’s age				
	Geographical working area	% working in northern Italy	% working in Southern Italy	0.043
		56.2	48.2	
	Work setting	% in hospital	% in university or private practice	0.002
		58.5	44.5	
Unable to perform NFE in >5 % of patients				
	Gender	% of males	% of females	0.003
		44.6	30.9	
	Age	% aged ≥50 years	% aged <50 years	0.001
		47.7	35.2	

Table 5 shows the significant associations between NFE methods and the demographic data. The choice of rigid endoscopes was only influenced by geographical working area as it was more frequent among the clinicians working in southern Italy (8.9 % *vs* 3.4 %; *p* = 0.003). Grading adenoidal hypertrophy on the basis of the standardised classifications was influenced by both geographical working area and the working setting: it was more frequent among clinicians working in southern Italy (54.6 % *vs* 42.8 %; *p* = 0.002) and those working in hospitals (51.7 % *vs* 42.1 %; *p* = 0.050), although logistic multivariate analysis only confirmed the significance of geographical working area adjusted for working setting (OR = 1.7, SE = 0.3; 95 % CI = 1.1–2.5; *p* = 0.006 for clinicians working in southern Italy).

**Table 5:** Methods of carrying out nasopharyngeal fiberendoscopy (NFE) in children by demographic variables (only statistically significant relationships)

Parameter	Demographic variables			*P*-value
Used to using rigid endoscopes	Geographical working area	% working in northern Italy	% working in southern Italy	0.003
		3.4	8.9	
Used to using endoscopes connected to a video recorder/monitor set	Gender	% of males	% of females	0.009
		78.5	68.0	
	Age	% aged ≥50 years	% aged <50 years	0.029
		80.4	73.5	
	Years since graduation	% graduated ≥30 years ago	% graduated <30 years ago	0.035
		80.0	73.2	
Used to using NFE bilaterally	Geographical working area	% working in northern Italy	% working in southern Italy	0.012
		40.1	49.8	
Used to using standardised classification to grade adenoidal hypertrophy	Geographical working area	% working in northern Italy	% working in southern Italy	0.002
		42.8	54.6	
	Work setting	% in hospital	% in university or private practice	0.050
		51.7	42.1	
Used to grading adenoidal hypertrophy according to Cassano’s classification [5]	Gender	% of males	% of females	0.047
		25.2	39.3	
	Age	% aged ≥50 years	% aged <50 years	<0.001
		17.5	35.4	
	Years since graduation	% graduated ≥30 years ago	% graduated <30 years ago	0.001
		19.5	35.5	
	Years since specialisation	% specialised for ≥20 years	% specialised for <20 years	0.024
		20.3	31.6	
	Geographical working area	% working in northern Italy	% working in southern Italy	0.001
		34.3	18.1	

None of the other demographic variables was statistically associated with attitudes towards NFE or the way in which it was carried out.

## Discussion

This is the first study specifically designed to evaluate Italian otolaryngologists’ attitudes towards using NFE to diagnose children, and the way in which do so in routine clinical practice. The randomised selection of the participants and the very small number who failed to respond makes it unlikely that the only respondents were otolarygologists who used NFE. Consequently, it is reasonable to believe that the study population was truly representative of otolaryngologists working in Italy and the members of the two most important Italian otolaryngological associations. The high response rate may have been partially due to the fact that the questionnaire was presented during our most important national congresses.

Despite some differences related to age, gender and geography, the data indicate that the majority of the respondents use NFE in their pediatric clinical practice and have a generally positive attitude towards it because nearly 70 % defined it as “a generally well-tolerated, minimally invasive examination that can be used in most children; very useful in clinical practice”. As NFE has only recently been considered the preferred means of diagnosing adenoidal hypertrophy in children [5–7], it is not surprising that younger otolaryngologists use it more frequently than those aged >50 years.

It is worth noting that more than one-third of the respondents (mainly females aged >50 years) declared that they used alternative means of diagnosing adenoidal disease, including a clinical evaluation (65 %), standardised questionnaires (19 %), posterior rhinoscopy (about 13 %), and nasopharyngeal radiography (about 3 %). This is not surprising because, until recently, the many proposed methods of assessing adenoid size were not very accurate. In particular, the most widely used clinical scores aimed of predicting the severity of nasal obstruction [9, 10] is the nasal obstruction index (NOI), which is based on the proportion of oral breathing and speech hyponasality [10]. This was proposed by Paradise as a reliable and reasonably valid means of detecting the presence and degree of adenoidal hypertrophy in 1998 [10], but we have shown that it alone is less accurate than NFE in predicting the rate of adenoidal obstruction in children with perceived obstructed nasal breathing or recurrent/chronic middle ear disease, and should therefore be abandoned [7, 11].

About 3 % of the responders said they used nasopharyngeal radiography as an alternative means of diagnosis, but it must be pointed out that its accuracy in assessing adenoidal hypertrophy (sensitivity 70 % and specificity 52 %) is much less than that of NFE [12]. Furthermore, it has been found that radiological measurements such as adenoidal thickness (the distance along a perpendicular line from the basiocciput to the adenoid convexity) and the adenoid-nasopharyngeal ratio (the ratio between adenoid thickness and the distance between the basiocciput and the posterior edge of the hard palate) do not correlate with obstructive symptom scores [8, 13].

However, some clinicians still advocate the use of radiological assessments in children with suspected adenoidal disease [2, 4, 14], especially in order to bridge the diagnostic gap in younger children who do not/cannot cooperate during an NFE examination [4]. Our findings indicate that there is some reluctance among Italian otolaryngologists to use NFE in small children, and most of them said that they only use it in older patients; only about 13 % (mainly ENT specialists working in hospitals in northern Italy) said they use NFE regardless of age. Furthermore, nearly one-third of the respondents (males aged >50 years) stated that they cannot complete an NFE examination in up to 25 % of patients. However, our experience [15] and that of others [3] indicates that NFE is feasible and tolerable in almost all children when it is carried out by a skilled otolaryngologist using a small-calibre flexible endoscope, and if every effort is made to find the best approach on the basis of the child’s age.

NFE is generally considered safe: almost all of the respondents said that fewer than 5 % of the procedures were associated with untoward side effects, mainly minor events such as nasal bleeding or traumatic lesions, and less frequently major events such as syncope (about 12 %) or desaturation (about 3 %). Thirty-eight percent excluded the possibility of using outpatient NFE in “children with a genetic syndrome” or “children aged <18 months in whom severe disease is highly suspected”. To the best of our knowledge, no specific guidelines have yet been published but, on the basis of our experience [15] and that of Pagella [3], we consider that NFE can generally be used in an outpatient setting even in young non-syndromic children.

In terms of the way in which is NFE is carried out, the responses to most of the items varied widely. The only items indicating almost unanimous agreement concerned the recommended type of endoscope (flexible), the position of the patient during NFE (sitting alone or on a parent’s lap), and restraint (with the parent blocking the movement of the child sitting on his/her lap). Various means of carrying out NFE examinations have been proposed [3, 4, 16–19], but our own experience confirms that the methods indicated by the answers of our respondents are effective and well-tolerated by almost all children [15]. Some authors [18–21] advocate the use of a rigid nasal endoscope, but this may be less well tolerated as it is associated with a failure rate of up to 12 % of children undergoing endoscopy in a supine position even after the administration of topical nasal anesthetics and decongestants [18, 19].

Only 32 % of our respondents said that they do not administer any topical drugs before an NFE examination, whereas the others pre-medicate the nasal cavities with local vasoconstrictors (30 %), an anesthetic (22 %), or lubricating ointment (about 16 %). We have previously reported that NFE can be successfully carried out in most patients without the aid of any of these [15] and, given that the use of topical decongestants has been proscribed by the Italian Medicines Agency in children aged <12 years [22], we suggest they should they not be used.

There was also considerable heterogeneity in the way that adenoidal hypertrophy is graded: just over half of the respondents base the grading on the percentage of choanal obstruction *and* the possible impaired patency of the Eustachian tube orifice, whereas about one-third only use the former. Only about 48 % said they used a standardised classification (mainly that of Cassano [5]), most of whom work in hospitals in southern Italy. This suggests the need for educational programmes supported by national otolaryngological societies aimed at promoting the use of standardised systems of scoring adenoidal hypertrophy in order to make medical reports comparable.

## Conclusions

The findings of this study seem to indicate that Italian otolaryngologists use NFE, and are quite confident about its effectiveness and safety in routine pediatric practice. However, there is some reluctance to using it in younger children, and no unanimous agreement about how it should be carried out. This lack of a standardised approach may account for failures in some patients, and encourage resort to alternative means of diagnosis that should actually be abandoned.

Given the medical advantages of NFE, it is essential to clarify the many still controversial aspects of the procedure by means of comparative studies and educational programmes supported by national health authorities.
